# Combined Liposuction and Excision of Lipomas: Long-Term Evaluation of a Large Sample of Patients

**DOI:** 10.1155/2015/625396

**Published:** 2015-01-28

**Authors:** Libby R. Copeland-Halperin, Vincenza Pimpinella, Michelle Copeland

**Affiliations:** ^1^Department of Surgery, Inova Fairfax Hospital, 3300 Gallows Road, Falls Church, VA 22042, USA; ^2^Division of Nursing, Mount Sinai Beth Israel Medical Center, 245 5th Avenue, New York, NY 10016, USA; ^3^Division of Plastic and Reconstructive Surgery, Icahn School of Medicine at Mount Sinai, 1001 5th Avenue, New York, NY 10028, USA

## Abstract

*Background*. Lipomas are benign tumors of mature fat cells. They can be removed by liposuction, yet this technique is seldom employed because of concerns that removal may be incomplete and recurrence may be more frequent than after conventional excision. *Objectives*. We assessed the short- and long-term clinical outcomes and recurrence of combined liposuction and limited surgical excision of subcutaneous lipomas. *Methods*. From 2003 to 2012, 25 patients with 48 lipomas were treated with liposuction followed by direct excision through the same incision to remove residual lipomatous tissue. Initial postoperative follow-up ranged from 1 week to 3 months, and long-term outcomes, complications, and recurrence were surveyed 1 to 10 years postoperatively. *Results*. Lipomas on the head, neck, trunk, and extremities ranged from 1 to 15 cm in diameter. Early postoperative hematoma and seromas were managed by aspiration. Among 23 survey respondents (92%), patients were uniformly pleased with the cosmetic results; none reported recurrent lipoma. *Conclusions*. The combination of liposuction and excision is a safe alternative for lipoma removal; malignancy and recurrence are uncommon. Liposuction performed through a small incision provides satisfactory aesthetic results in most cases. Once reduced in size, residual lipomatous and capsular tissue can be removed without expanding the incision. These favorable outcomes support wider application of this technique in appropriate cases.

## 1. Introduction

Lipomas are common, benign soft tissue tumors that occur on the body surface either sporadically or in association with inherited disorders of fat metabolism. They are typically painless and mobile and enlarge slowly. Histologically, they consist of enlarged adipocytes with uniform nuclei and are usually surrounded by a fibrous capsule [1]. They are aesthetically unpleasing and can become irritated or infected. Surgical intervention is typically performed when the lipoma is uncomfortable, limits function, or otherwise bothers the patient. Several methods have been described for removal, including direct excision, excision through a remote incision [2], liposuction [3, 4], endoscopic excision [5], and laser extirpation [6]. Although small lipomas (up to 3 cm in diameter) can usually be excised directly, excision of large lipomas can be associated with more extensive scarring. Suctioning prior to excision to debulk the lipoma reduces the size of the incision and resulting scar. For lipomas of intermediate (4–10 cm) or large (>10 cm) size [7], liposuction can improve the early cosmetic result, minimize operative time, and reduce the risk of postoperative hematoma and seroma formation [4, 8].

Although there have been multiple reports of successful liposuction for lipoma removal, the technique is not widely embraced. Objectors argue that liposuction limits visualization of the tumor, fragments the specimen confounding histopathological examination for features of malignancy, and leaves residual lipomatous or capsular tissue that predisposes to recurrence [5, 7, 9].

We describe the largest series of lipomas removed through a combination of liposuction and direct excision and report patient outcomes over a decade to address concerns about recurrence and malignancy.

## 2. Methods

For 25 consecutive patients with superficial fat tumors typical of lipomas seeking excision, we offered two alternative techniques for removal: direct excision alone or a combination of liposuction and direct excision. Patients were advised that, should fluid accumulate following excision, serial aspiration might be required with either method. After discussion of the potential benefits, limitations, and risks of each technique, all patients chose the liposuction and excision combination approach.

### 2.1. Technique of Removal

The lipomatous mass was outlined in its entirety and infiltrated with a solution 1% xylocaine with epinephrine, 1 : 100,000 for local anesthesia and to promote hemostasis. A 3 mm sharp liposuction cannula was then inserted through a 1 cm incision made in the midportion of the surface of the mass with a #15 scalpel blade. The bulk of the lipoma was removed by aspiration before removing residual tissue and capsule by direct, sharp, and step-wide excision. All extracted specimens were submitted for histopathological examination to exclude liposarcoma or atypical cells. The wound was then irrigated and assessed for hemostasis. Closure was achieved with subcutaneous 5-0 Vicryl and Monocryl sutures without drain placement, Steri-strips were applied, and a bulky dressing was secured. After suctioning of lipomas from the back or abdomen, compressive garments were used to secure the bulkier dressings.

### 2.2. Postprocedural Management

While drain insertion after removal of large lipomas is reasonable, serial aspiration avoids the need for additive drainage scars or elongation of the incision and is preferred to control postoperative fluid accumulation. The dressing was changed 1 week postoperatively and topical silicone gel and pigment-reducing cream were applied for several weeks to reduce scar thickening, retraction, or discoloration. The incision was evaluated postoperatively by the senior surgeon to assess healing and the aesthetic result.

### 2.3. Late Follow-Up

Follow-up questionnaires were sent to all patients in 2013 (1–10 years postoperatively) to collect retrospective data about the quality and durability of the result, late complications, further treatment, or development of additional lipomas (see appendix).

## 3. Results

Between 2003 and 2012, 48 lipomas were removed by combined liposuction and excision from 25 patients (17 women and 8 men), ranging in age from 19 to 77 years (mean 49.8 years). Six had multiple lipomas and 19 had solitary masses. Lipomas ranged in diameter from 1 to 15 cm (mean 5.4 cm); 7 were smaller than 3 cm. Two were located on the head or neck, 11 on the back, 2 on the abdomen, 31 on the extremities, and 2 on the groin (see Table 1). In one case, liposuction was used to reduce the volume of a diffuse lipoma following which the capsule was removed by direct excision.

### 3.1. Pathological Examination

All extracted and excised specimens submitted for histopathological evaluation were sufficient for analysis and had characteristics of benign lipomas; none contained morphologically dysplastic or malignant cells.

### 3.2. Early Postoperative Follow-Up

**Figure 1:**
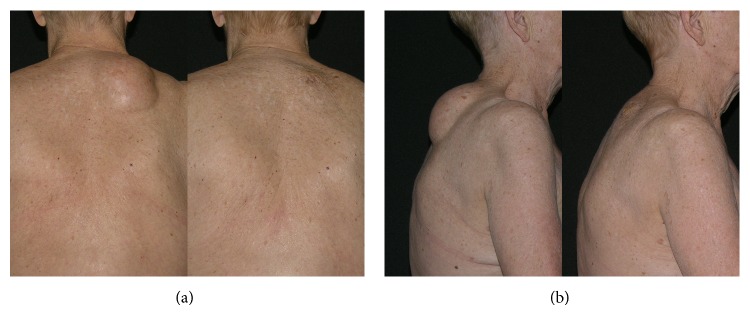
Pre- and 1-month postoperative photographs of an 80-year-old woman with 15 × 13 cm lipoma. (a) Back view and (b) right lateral view.

During early follow-up 1 to 12 weeks postoperatively, repeated aspiration was required in 18 cases with eventual resolution, including one hematoma after removal of a 10 cm abdominal lipoma and one seroma after removal of a 15 cm lipoma from the back (see Figure 1).

### 3.3. Long-Term Outcomes

Later outcomes were assessed by written responses to a survey, to which 23 patients responded (92%) 4 months to 10 years postoperatively (mean 7 years; median 6.5 years); two patients did not respond. None of the respondents identified complications of the procedure or recurrence of lipoma, appearance of new lipomas, hyperpigmentation, scarring, or clinical evidence of malignant transformation.

## 4. Discussion

Since its introduction in 1975 by Fischer, followed by Illouz's “wet technique” in 1977 [9], the indications for liposuction have expanded to include lipodystrophy, gynecomastia, and evacuation of lipomas [10–14]. Removal of lipomas by this technique to decrease incision size and scarring was described in 1990. Al-Basti and El-Khatib [15] followed liposuction by capsular excision through the cannula incision, and Choi et al. [16] used tumescent liposuction to remove lipomas. Despite reports of favorable experiences, surgeons often forego liposuction out of concern that incomplete removal or recurrence might compromise outcomes or that cellular disruption might impede histopathological examination or mask malignant features.

The combined liposuction and excision technique facilitates complete removal of lipomas through small incisions. Fibrous lipomas and angiolipomas are less amenable to liposuction; others have indistinct borders or transitions to nonlipomatous adipose tissue. While these require greater direct excision of the fibrous components, initial liposuction aided debulking and facilitated removal through smaller incisions. Early postoperative fluid accumulation developed in over a third of cases (incidence 37.5%) but responded to percutaneous aspiration without residua. Postoperative hematoma or seroma might have been avoided by placement of conventional drains, which would entail additional scarring, as discussed with patients preoperatively. There was no clinical recurrence among the 23 patients we queried after a median postoperative interval of 6.5 years. The local recurrence rate of lipomas after surgical excision has been reported as 1-2% over an indefinite period [17]; hence while a larger sample of patients is required to establish more precisely the rate when liposuction is initially employed, the long-term durability of the procedures appears comparable. Caution is appropriate in applying this technique in patients with single or multiple small lipomas in the same body region, as occurs in cases of multiple familial lipomatosis, because disrupting the individual capsules of these lipomas can be ultimately more traumatic to surrounding tissue than conventional excision.

None of the lesions in this series had clinical features suggestive of liposarcoma, hibernoma, or lipoblastoma. Liposarcomas typically occur between the 5th and 7th decades of life in the deeper soft tissues of the extremities [18]. Several studies have demonstrated preservation of adipocyte integrity after liposuction [19, 20]. Histopathologic examination of the specimens in this series was not hampered, and none identified malignancy. A particular concern with the combined liposuction/excision method is the potential risk of disseminating malignant cells upon capsular disruption. Although liposarcomas account for approximately 20% of all soft tissue sarcomas in adults [17], their population incidence is relatively low (approximately 2.5 cases per million annually) [21]. Before utilizing liposuction for lipoma, therefore, the surgeon should assure the absence of clinical features associated with liposarcoma, including rapid growth, pain, or immobility of the soft tissue tumor. When liposarcoma is suspected, biopsy is essential, coupled with ultrasound examination prior to complete excision.

This study is limited by sample size, which is insufficient to identify recurrence rates less than about 2 percent. None of the lipomas had malignant features, and we caution clinicians to carefully assess soft tissue tumors for atypical clinical features before employing the intervention we describe. Despite these limitations, our observations suggest that the combination of liposuction and excision is a safe option for removal of subcutaneous lipomas that yields successful results. Outcomes could differ for submuscular lipomas. While a questionnaire is not entirely sufficient for evaluating recurrence, it provides a subjective method of assessing whether the patient detects recurrence. The outcomes in the two patients who failed to respond to the survey could not be determined.

Since the management of lipomas is inherently conservative, excision is recommended only when the tumors are symptomatic because of their size or location, have suspicious clinical features, or are cosmetically unacceptable to the patient, and the incidence of malignancy is low; we believe that removal by combined liposuction and direct excision is a reasonable alternative to direct, open excision.

The use of liposuction permits a smaller incision and favorable aesthetic results, without exposing patients to recurrence or compromising pathological analysis in the vast majority of cases. A randomized trial comparing liposuction with conventional direct excision is necessary to more conclusively compare the outcomes of these techniques.

## Figures and Tables

**Table 1:** Summary of patients and lipomas.

Patient	Age (years)	Lesion diameter (cm)	Location	Initial follow-up (weeks)	Long-term follow-up (years)
(1) S. R.	44	5 cm; 7 cm	RT shoulder; LT flank	3	10

(2) M. S.	50	7 cm	LT shoulder	1	10

(3) C. T.	55	5 cm	LT arm	1	10

(4) C. K.	40	10 cm	RT back	4	9

(5) G. G.	77	15 cm	Upper back	8	8

(6) A. C.	65	10 cm	RT posterior knee	1	8

(7) P. T.	24	6 cm	Back	1	7

(8) H. F.	49	10 cm	Upper back	3	7

(9) A. T.	19	10 cm	RT ankle	52	7

(10) D. S.	52	7 cm	RT back	4	7

(11) R. M.	46	7 cm	Upper back	none noted	N/A

(12) S. A.	54	10 cm	Upper back	2	7

(13) R. T.	59	2 cm	RT temple	4	6

(14) A. K.	52	4 cm	LT mid back	1	5

(15) A. I.	42	3 cm; 4 cm; 5 cm	RT upper back; RT lower back; LT jawline	2	N/A

(16) C. L.	53	2–5 cm	RT lower lateral thigh; RT midlateral thigh; RT upper medial thigh; RT middle medial thigh; RT lower medial thigh; HIP; RT upper buttock; RT lower buttock; RT outer thigh	6	5

(17) N. P.	46	13 cm	Upper back	8	5

(18) C. L.	53	2–5 cm	LT buttock; LT infragluteal fold; LT midlateral exterior thigh; LT interior thigh; LT arm	6	5

(19) C. L.	53	2–5 cm	LT upper forearm; LT lower forearm; LT inner thigh; LT upper outer thigh; LT medial thigh, LT lower thigh; LT upper anterior thigh; LT medial thigh; LT lower anterior thigh	6	5

(20) L. Q.	42	10 cm	LT lower abdomen	12	5

(21) A. S.	50	10 cm	RT rectal-vaginal	5	5

(22) J. Z.	62	10 cm	RT arm	1	3

(23) S. I.	60	7 cm	RT shoulder	12	1

(24) M. A.	47	1.5 cm; 3 cm	RT upper elbow; LT groin	1	1

(25) A.D.	80	15 cm	RT upper back	4	1

## Appendix

### Lipoma Follow-Up Questionnaire

1. Did your lipoma return or did you need to seek further treatment for your lipoma? yes or no
2. If so, what year?
3. Did you have any postoperative bruising or skin dimpling at the surgical site? yes or no
4. Have you had other lipomas removed before or after this one? yes or no
5. If so, what year and how did your postoperative recovery compare (pain, bruising, etc.)?
6. Do you have a history of cancer? yes or no
7. Do you have any other concerns about the procedure or your recovery?
